# Stroke and Disseminated Intravascular Coagulation in a Patient With Metastatic Prostate Cancer

**DOI:** 10.1177/19418744231172622

**Published:** 2023-04-27

**Authors:** Jonah P. Zuflacht, Joshua M. Levine

**Affiliations:** 1 Department of Neurology, 21798 Hospital of the University of Pennsylvania, Philadelphia, PA, USA; 2 Neurology, Neurosurgery, and Anesthesiology and Critical Care, 6572 University of Pennsylvania, Philadelphia, PA, USA

**Keywords:** cerebrovascular disorders, clinical specialty, hematology, intracranial hemorrhages, neurocritical care, stroke

## Abstract

Cancer and stroke comprise two of the most common causes of death worldwide. Despite a significantly increased risk of stroke among patients with cancer, most stroke trials have excluded patients with malignancy. There is thus limited evidence to help guide management decisions in this complex population. We present the case of a 78-year-old man with recurrent strokes – both ischemic and hemorrhagic – in the setting of newly-identified metastatic prostate cancer. An atypical cause of cancer-associated stroke is reviewed and the management is discussed.

## Case Presentation – Part 1

A 78-year-old man with hypertension, who was systemically anticoagulated with rivaroxaban for atrial fibrillation, presented to our emergency room four days after the sudden onset of impaired vision on the right side. He reported no weakness, numbness, dizziness, or difficulty with language.

**Figure 1.**
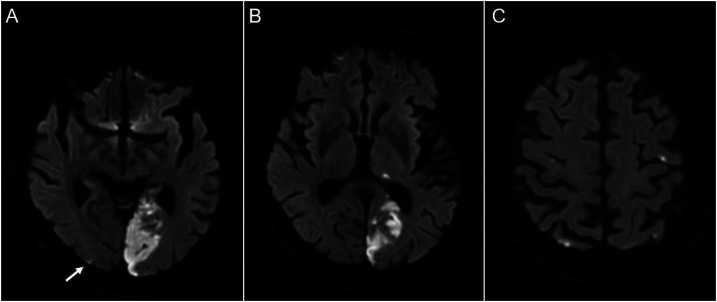
Brain MRI upon initial presentation. Large left PCA territory stroke as evidenced by restricted diffusion (A and B). Scattered, smaller infarcts are seen in the bilateral MCA territories (C). An additional punctate focus of restricted diffusion is seen in the right MCA/PCA territory (A, arrow).

Upon initial evaluation, the patient had a right homonymous hemianopia and no other neurological deficits. Laboratory studies were notable for new mild thrombocytopenia (platelet count was 91 × 10^3^/μl, down from 277 × 10^3^/μl one year prior, reference range: 150 – 400 × 10^3^/μl), elevated international normalized ratio (INR, 1.3), elevated partial thromboplastin time (PTT, 29.9), and elevated D-dimer (24.40 µg/ml fibrinogen equivalent units, reference range: .00 – .50 FEU). The rivaroxaban level, as assessed via anti-Xa activity, was not measured. MRI of the brain without contrast revealed areas of restricted diffusion in the left occipital lobe and dorsal thalamus consistent with subacute infarctions in the posterior cerebral artery (PCA) distribution (Figures 1A, 1B). There were additional punctate foci of restricted diffusion in both middle cerebral artery (MCA) territories (Figure 1C). CT angiography of the brain showed no vascular abnormalities and a transthoracic echocardiogram revealed no cardiac source of embolism.

### Reasoning

Though atrial fibrillation may result in a modest elevation of the D-dimer, the degree of D-dimer elevation should prompt evaluation for occult malignancy. A CT of the torso showed heterogeneous enhancement of the right prostate gland concerning for prostate cancer, and several enlarged mesenteric and periaortic lymph nodes suspicious for metastases. Prostate specific antigen (PSA) was markedly elevated (654.762 ng/mL, reference range: 0 - 4 ng/mL). It was recommended that the patient be transitioned from rivaroxaban to enoxaparin. For unclear reasons, however, the patient was discharged home on apixaban and arrangements were made for further evaluation of his prostate cancer by a urologist.

Prostate cancer is one of the most common malignancies in adult men. Like patients with other carcinomas, such as breast or lung, patients with prostate carcinoma are at increased risk of venous thromboembolic events.^1^ This hypercoagulable milieu is attributable to both the prothrombotic effects of the tumor itself (via release of tissue factor and cancer pro-coagulant) as well as the overall inflammatory state.^2^ Patients receiving hormonal therapy as part of their treatment regimen appear to be at even higher risk.^1,2^

Whether or not there is an increased risk of ischemic stroke in patients with untreated prostate cancer is less understood. Mucin-secreting tumors, including adenocarcinoma of the prostate, are thought to promote platelet-rich thrombi formation and thus an increased risk of stroke.^3^ Yet high quality prospective data are lacking, and a large population-based study from Sweden suggests that untreated patients – while at increased risk of both deep vein thrombosis and pulmonary embolism – do not carry an increased risk of arterial embolic events.^4^

In addition to the symptomatic left PCA territory infarct, our patient was found to have scattered infarcts in the bilateral MCA territories (Figure 1). This imaging pattern – with multiple infarcts in multiple vascular territories – is strongly suggestive of underlying malignancy.^5^ Though it is possible that atrial fibrillation may have also contributed, there was no history of rivaroxaban non-adherence. Furthermore, a significantly elevated D-dimer is highly characteristic of cancer-related stroke (but uncharacteristic of atrial fibrillation).^5^

## Case Presentation – Part 2

**Figure 2.**
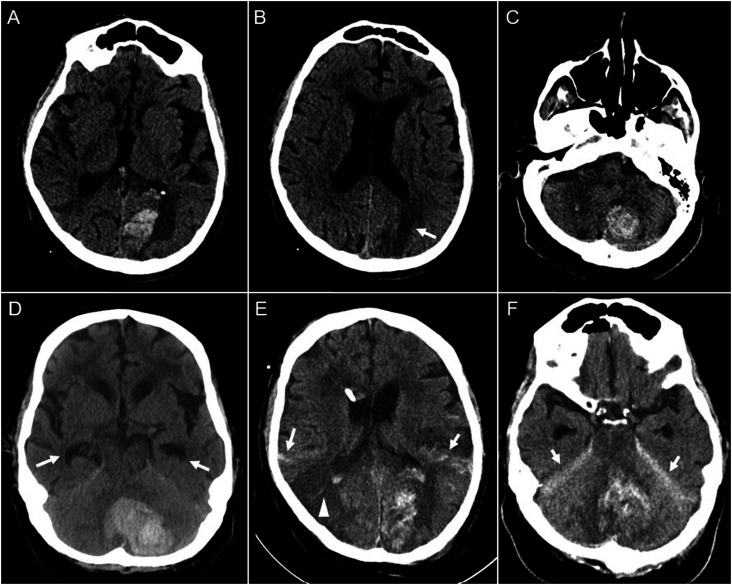
Serial CT head images after representing three weeks later. Initial CT head notable for hemorrhagic conversion of subacute left PCA stroke (A), new left parietal infarct (B, arrow), and left greater than right bilateral cerebellar hemorrhages (C). Repeat CT head obtained after clinical deterioration shows further expansion of the left cerebellar hemorrhage (D) with associated hydrocephalus as evidenced by enlargement of temporal horns (arrows). CT head after sub-occipital craniectomy and placement of EVD shows new right temporal-occipital ischemic stroke (E, arrowhead) as well as bilateral subarachnoid hemorrhage (E, arrows) and tentorial subdural hematomas (F, arrows).

Three weeks later the patient presented to an outside hospital emergency department with sudden onset headache and difficulty walking. He was somnolent, only opening his eyes briefly to voice, and was able to follow commands. He had a right eyelid ptosis and a right homonymous hemianopia, and a right hemiparesis. The platelet count was 78 × 10^-3^/ul and INR was 1.6. CT scan of the head showed an acute left occipital hemorrhage concerning for hemorrhagic transformation of a left PCA-territory ischemic stroke (Figure 2A), an acute infarct in the left parietal lobe (Figure 2B), and bilateral (left greater than right) cerebellar hemorrhages (Figure 2C).

### Reasoning

This patient initially presented with a left PCA ischemic stroke despite therapeutic anticoagulation with rivaroxaban. A markedly elevated D-dimer prompted screening for occult malignancy and subsequently lead to the diagnosis of metastatic prostate cancer. Three weeks later, he represented with multifocal supra- and infra-tentorial intracranial hemorrhages as well as new ischemic strokes. This clinical picture, together with the combination of thrombocytopenia and INR elevation, was suggestive of disseminated intravascular coagulation (DIC).

The etiology of our patient’s initial ischemic strokes may also have been DIC. In retrospect, though this patient went on to have both overt clinical and laboratory evidence of DIC, the mild thrombocytopenia during his initial evaluation might have been an early diagnostic clue.

DIC is the most common coagulopathy in patients with prostate cancer. Roughly 13% of patients with metastatic disease develop DIC, and nearly 25% of all DIC cases are related to metastatic prostate carcinoma.^6^ DIC is an acquired syndrome defined by the pathological activation of the coagulation system.^7^ Frequently associated with sepsis and the systemic inflammatory response syndrome, DIC may be triggered by a variety of underlying disease processes including trauma, liver failure, obstetric complications, and cancer.^7^ Identification of one of these underlying disorders is critical in establishing the diagnosis of DIC according to the diagnostic algorithm proposed by The International Society on Thrombosis and Haemostasis (ISTH). Laboratory criteria involved in this scoring system include the degree of thrombocytopenia, elevation of the D-dimer, prolongation of the prothrombin time, and reduction in fibrinogen.^8^

**Figure 3.**
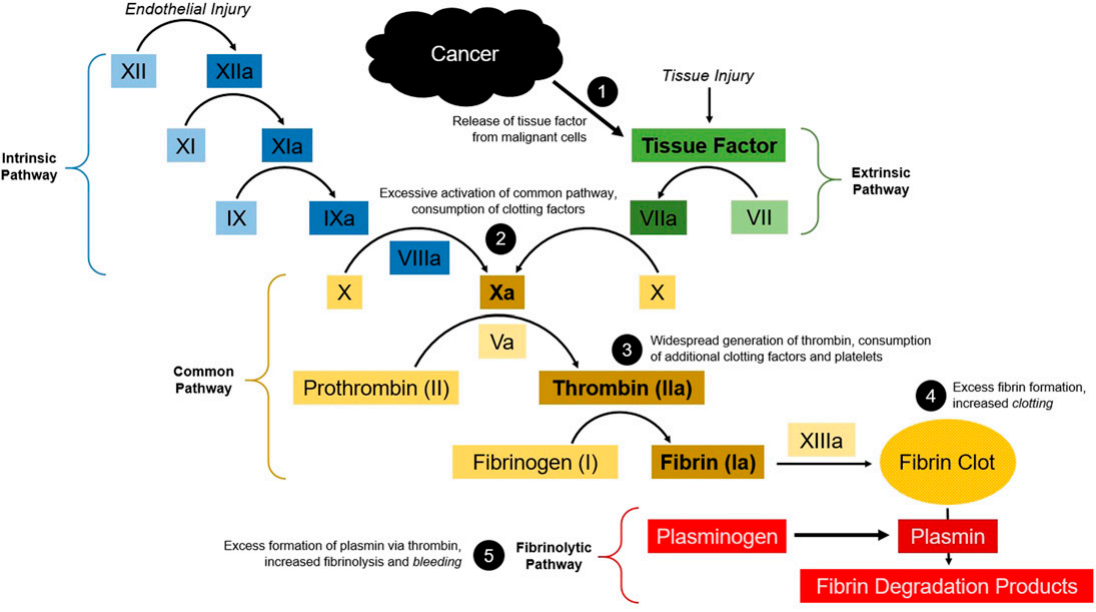
Disruption of the coagulation cascade in the setting of cancer-related disseminated intravascular coagulation. Massive release of tissue factor from malignant cells causes activation of the extrinsic pathway (1). This leads to excessive activation of the common pathway and consumption of clotting factors (2). The result is widespread generation of thrombin, consumption of additional clotting factors and platelets (3) leading to both excessive clotting (4) and bleeding. Thrombin also acts to convert plasminogen to plasmin, thereby accelerating the fibrinolytic pathway (5) and further increasing the risk of bleeding.

The common pathophysiological mechanism in DIC is widespread thrombin generation which results in activation of the clotting cascade and derangement of the laboratory studies referenced above.^7,9^ Among patients with cancer, this is likely mediated by the expression of procoagulant substances such as tissue factor (Figure 3).^7^

A chronic form of DIC, characterized by a mild decrease in clotting factors and platelets with insidious thrombin formation, is more common in patients with prostate cancer and is often subclinical.^6^ However, acute DIC – characterized by massive fibrin generation, impaired fibrinolysis, and rapid consumption of clotting factors – may also occur and can lead to severe neurologic complications.^6^

## Case Presentation – Part 3

He was treated emergently with 25 U/kg of prothrombin complex concentrate (PCC) and transferred to the neurological intensive care unit at our hospital. While the patient’s neurological exam did not change, a repeat CT scan of the head six hours later showed interval enlargement of the left cerebellar hemorrhage with increased mass effect and effacement of the fourth ventricle.

Hours later the patient became obtunded and developed a forced downward gaze deviation. The INR was now 2.0 and the platelet count was 89 × 10^−3^/ul. A repeat CT scan of the head showed continued expansion of the left cerebellar hemorrhage and new non-communicating hydrocephalus (Figure 2D). He was given 1 unit of platelets and an additional 25 U/kg of PCC before being brought to the operating room for decompressive sub-occipital craniectomy and placement of an external ventricular drain. During surgery there was significant intracranial bleeding and the patient was given 2 more units of platelets, 1 unit of fresh frozen plasma, and 1 unit of packed red blood cells. He also received andexanet alfa.

Over the ensuing twenty-four hours, the patient developed bleeding from multiple locations including the oropharynx, urethra, rectum, and intravenous catheter insertion sites. Serial laboratory studies revealed worsening coagulopathy with INR of 2.7 and fibrinogen of 83 mg/dL, anemia with nadir hemoglobin of 6.6 *g*/dL, and thrombocytopenia with nadir platelet count of 68 × 10^3^/ μl. The clinical picture was consistent with fulminant DIC. Despite aggressive efforts to correct these laboratory abnormalities, the patient accrued new ischemic strokes as well as subarachnoid and subdural hemorrhages (Figures 2E, 2F). The patient died after his family changed the goals of care to comfort-measures only.

### Reasoning

Despite maximal medical therapy and neurosurgical interventions, our patient died less than one month after first seeking neurological evaluation. It is possible that the fulminant course observed in our patient was precipitated by anticoagulation-associated intracerebral hemorrhages, due to the evolution of a previously quiescent form of DIC related to underlying prostate cancer, or a combination of the two.

The neurological manifestations of DIC are defined by thrombotic (anti-fibrinolytic) and hemorrhagic (fibrinolytic) phenotypes. While one form may predominate, it is likely that – on a molecular level – both processes occur simultaneously.^8^ As a result, patients may present with ischemic stroke, intracerebral hemorrhages, or both. Patients with prostate cancer may have a higher incidence of intracerebral bleeding compared to patients with other solid tumors.^10^

## Discussion and Management

In 2023, an estimated 1,958,310 new cancer cases and 609,820 cancer deaths will occur in the United States. After two decades of decline, the incidence of prostate cancer appears to be increasing.^11^ Among patients with cancer, the risk of stroke is more than twice that of the general population.^12^ Yet most stroke trials have excluded patients with active malignancy and there is thus limited evidence to help guide treatment decisions in this complex population.

Disseminated intravascular coagulation (DIC), an acquired syndrome defined by the pathological activation of intravascular clotting, is the most common coagulopathy in patients with prostate cancer. Both insidious (more common) and fulminant (less common) presentation occur. The diagnosis of DIC, according to criteria proposed by the ISTH, is predicated upon identification of an underlying cause. Laboratory studies including the degree of thrombocytopenia, elevation of the D-dimer, prolongation of the prothrombin time, and reduction in fibrinogen are key factors in this diagnostic algorithm.

Vascular neurologists and neuro-intensivists should be aware of the neurological complications of DIC. Patients may present with ischemic strokes, intracerebral hemorrhages, or both. The clinical presentation is defined by whether the thrombotic (anti-fibrinolytic) or hemorrhagic (fibrinolytic) form predominates.

Treatment of DIC is predicated upon treatment of the underlying cause. Blood pressure and ventilator support are often necessary, particularly in overt cases. Blood products – such as fresh frozen plasma, cryoprecipitate, and platelets may help minimize the risk of hematoma expansion in those with intracerebral hemorrhage or other serious bleeding complications and mitigate intraoperative bleeding in those undergoing surgery.^8^

Our patient initially received PCC in attempt to reverse the coagulopathy associated with apixaban; there was no suspicion for DIC at the time of PCC administration. Indeed, PCC is typically avoided in patients with DIC given concern for provoking additional thrombotic complications. Andexanet alpha was unavailable at the outside hospital and is not clearly superior to conventional reversal strategies.

The ongoing ANNEXA-1 study – a randomized, controlled clinical trial evaluating the efficacy and safety of andexanet alfa compared to usual care in patients with intracranial hemorrhage anticoagulated with a direct oral anticoagulant – should help clarify the preferred approach in this patient population.^13^ While he ultimately received andexanet alpha in the operating room, it is unclear whether this had any beneficial effect given the principal driver of his bleeding (DIC) and considering the amount of time since his last apixaban dose (>24 hours). Furthermore, there is a risk of catastrophic thrombotic events in patients receiving the combination of PCC and andexanet alpha.^14^
